# Screening for the Proteins That Can Interact with Grouper Nervous Necrosis Virus Capsid Protein

**DOI:** 10.3390/v12090985

**Published:** 2020-09-04

**Authors:** Po-Yu Huang, Han-Chia Hsiao, Szu-Wen Wang, Shao-Fu Lo, Ming-Wei Lu, Li-Li Chen

**Affiliations:** 1 Center of Excellence for the Oceans, National Taiwan Ocean University, No. 2, Pei-Ning Road, Keelung 20224, Taiwan; abcm1042@hotmail.com; 2 Institute of Marine Biology, National Taiwan Ocean University, No. 2, Pei-Ning Road, Keelung 20224, Taiwan; elly010042@gmail.com (H.-C.H.); wen810722@hotmail.com (S.-W.W.); john81john81@yahoo.com.tw (S.-F.L.); 3 Department of Aquaculture, National Taiwan Ocean University, No. 2, Pei-Ning Road, Keelung 20224, Taiwan; mingwei@mail.ntou.edu.tw

**Keywords:** nervous necrosis virus, immunoprecipitation assay, protein–protein interaction, creatine kinase, far-Western blot

## Abstract

Nervous necrosis virus (NNV) can infect many species of fish and has an 80–100% mortality rate. NNV capsid protein (NNVCP) is the only structural protein of NNV, but there are few studies on the protein–protein interaction between NNVCP and the host cell. To investigate NNV morphogenesis, native NNV capsid protein (NNVCP) was used to screen for protein–protein interactions in this study. The results identified that 49 grouper optic nerve proteins can interact with NNVCP and may function as putative receptor or co-receptor, cytoskeleton, glucose metabolism and ATP generation, immunity, mitochondrial ion regulation, and ribosomal proteins. Creatine kinase B-type (CKB) is one of those 49 optic nerve proteins. CKB, a kind of enzyme of ATP generation, was confirmed to interact with NNVCP by far-Western blot and showed to colocalize with NNVCP in GF-1 cells. Compared to the control, the expression of CKB was significantly induced in the brain and eyes infected with NNV. Moreover, the amount of replication of NNV is relatively high in cells expressing CKB. In addition to providing the database of proteins that can interact with NNVCP for subsequent analysis, the results of this research also verified that CKB plays an important role in the morphogenesis of NNV.

## 1. Introduction

Grouper is an aquaculture fish with substantial economic value and it is an important source of income in many Asian countries. Like other high-density aquaculture species, groupers are also threatened by many pathogens, including *Vibrio alginolyticus*, iridovirus, and nervous necrosis virus (NNV) [1,2,3,4]. NNV is highly lethal to all grouper stages and results in massive economic losses worldwide [5]. NNV is the causative agent of viral nervous necrosis (VNN) disease, which has a wide host range, infecting at least 40 families of fish species [6,7,8,9,10]. NNV infects the central nervous system and causes vacuolation of the brain and retina [11]. NNV has a non-enveloped icosahedral structure and belongs to the family Nodaviridae (genus *Betanodavirus*), and its genome consists of two single-stranded positive-sense RNAs. RNA1 encodes the RNA-dependent RNA polymerase (RdRp), and RNA2 encodes the viral capsid protein [12,13,14,15,16]. The NNV capsid protein (NNVCP) is the only structural protein of the virion and has been shown to determine the host range [17]. Betanodaviruses have been classified into the following four genotypes based on the sequence of the RNA2 segment: tiger puffer NNV (TPNNV), striped jacked NNV (SJNNV), red-spotted grouper NNV (RGNNV), and barfin flounder NNV (BFNNV) [18]. Despite its relatively simple virion structure, no clear NNV receptor has been identified, and the mechanism of NNV infection remains unclear.

It is very difficult to prevent NNV in the aquaculture process except through preventing infection by parental fish, ensuring water quality, and actively managing feed and livestock. NNV is very contagious, and it is difficult to completely eliminate NNV from aquaculture environments. DNA vaccines, interferons, or immunostimulants can be used to reduce NNV infections and outbreaks, but they are not sufficient to completely control the epidemic [19,20,21,22]. However, the functional roles of host factors interacting with the NNVCP in viral genome replication remain ambiguous.

Although NNV can infect more than 30 marine and freshwater fish species, and furthermore, NNV capsid protein (NNVCP) was the only external viral structural protein in virus particles, to date, only GHSC70 and MmHSP90ab1 have been identified as attachment receptors for NNV [23,24]. However, preventing these proteins from interacting with cells cannot inhibit viral infection, so NNV is thought to still have other undiscovered host receptors or co-receptors. In addition, many cellular proteins that may be involved in the NNV morphogenesis have not been verified. Thus, native NNVCP isolated from the grouper cell line GF-1, rather than a recombinant protein generated by bacteria, was applied in this study to investigate the proteins that can interact with NNVCP. Optic nerves were selected from uninfected orange-spotted grouper (*Epinephelus coioides*) to evaluate protein interactions with native NNVCP by immunoprecipitation (IP). We annotated the proteins using 1DLC, LTQ-Orbitrap MS database of the *Epinephelus genus* in NCBI and UniProt. The 49 identified proteins could contribute as putative receptor or co-receptor, cytoskeleton, glucose metabolism, and ATP generation, immunity, mitochondrial ion regulation, and ribosomal proteins. 

The virus uses the energy of the host cell to complete its morphogenesis. In the early stage of NNV infection, viral replication leads the host cell to rapidly deplete ATP; then, NNV uses grouper voltage-dependent anion channel 2 (GVDAC2) to maintain the sufficient ATP for NNV RNA synthesis [25]. Since there are many energy-generating mechanisms in cells, in this study, we investigated the role of creatine kinase (CK), another molecule that catalyzes energy production, during NNV infection. Far-Western blot and cellular colocalization analysis confirmed that creatine kinase brain type (CKB) can interact with NNVCP, and it was observed that CKB can enhance NNV replication in GF-1 cells. In addition to providing more information about the types of cellular proteins that can bind to NNV, this study also confirmed that enzymes that contribute to cellular ATP generation, such as CK, provide assistance in the morphogenesis of NNV.

## 2. Materials and Methods

### 2.1. Cell Line and Cell Transfection

The grouper fin cell line (GF-1, BCRC 960094) was obtained from the Bioresource Collection and Research Center (BCRC) in Taiwan. Cell culture was performed according to the work of Chang and Chi [23]. Briefly, cells were cultured in Leibovitz’s L-15 medium (Thermo Fisher Scientific Inc., Waltham, MA, USA) with 5% fetal bovine serum (FBS) (Thermo Fisher Scientific) and maintained at 27 °C. Transfection was performed in a 24-well format. About 1 × 10^5^ cells were seeded in each well of the 24-well culture plate and cultured overnight. The CK B-type was amplified from orange-spotted groupers (*Epinephelus coioides*) optic nerve cDNA with the following primer set CKB-F/CKB-R (5′-CAGGGGGATCCCATGCCTTTCGGTAA-3′/GTCAGGGAATTCTTACTTCTGGGCGGG, the underlined bases indicate the *Bam*HI and *Eco*RI restriction sites for gene cloning). The resultant recombinant plasmid, pcDNA3-CKB plasmid, was mixed with Lipofectamine 2000 kit (Thermo Fisher Scientific) in the medium and laid over the cultured cells according to the manufacturer’s recommendations. Two days after transfection, the cells were cultured in selective medium contained with geneticin (G418) (Thermo Fisher Scientific) (400 μg/mL). The selective medium was replaced every three days.

### 2.2. Expression and Purification of Recombinant NNVCP and CK B-type, and Antibody Preparation

Full-length recombinant NNVCP (rNNVCP) and CK B-type (rCKB) were generated using the pET28b+ system. The sequence encoding NNVCP protein was amplified from NNV cDNA with the following primer set: NNVCP-F/NNVCP-R (5′-CGAATTCATGGTACGCAAAGGTGAAAAGA-3′/5′-ATAGTCGACTTAGTTTTCCGAGTCAACCCT-3′, the underlined bases indicate the *Eco*RI and *Sal*I restriction sites for gene cloning); the CK B-type was amplified from orange-spotted groupers optic nerve cDNA with the following primer set: CKB-F/CKB-R (5′-CAGGGGGATCCCATGCCTTTCGGTAA-3′/GTCAGGGAATTCTTACTTCTGGGCGGG), the underlined bases indicate the *Bam*HI and *Eco*RI restriction sites for gene cloning). The resultant recombinant plasmid, pET28b-NNVCP and pET28b-CKB, was transformed into the *Escherichia coli* BL21 (DE3) strain. *E. coli* BL21 (DE3) cells were cultured in LB medium with 25 μg/mL kanamycin, and the protein was induced with 1 mM isopropyl-β-d-thiogalactopyranoside (IPTG). Recombinant proteins tagged with six consecutive histidines were purified by QIAexpressionist nickel-nitrilotriacetic acid (Ni-NTA) metal-affinity chromatography (Qiagen, Hilden, Germany) according to the manufacturer’s recommendations. The resins were washed with buffer (pH 8.0) containing 50 mM sodium phosphate, 0.3 M sodium chloride and 10 mM imidazole and eluted with buffer (pH 8.0) containing 50 mM sodium phosphate, 0.3 M sodium chloride, and 250 mM imidazole. The eluted protein was then concentrated using Amicon Ultra-15 centrifugal filters (Merck Millipore, Burlington, MA, USA) in PBS buffer and stored at 4 °C for subsequent antiserum production.

Rats were selected to produce the NNVCP-specific polyclonal antibodies. In brief, rats were hyperimmunized by injection with 250 μg rNNVCP proteins emulsified in complete Freund’s adjuvant. Subsequent booster injections were carried out with 250 μg protein emulsified in incomplete Freund’s adjuvant every two weeks. Antisera were collected after the antibody titer had peaked. 

### 2.3. Virus Infection 

NNV was isolated from grouper larvae with NNV disease. NNV was propagated in GF-1 cells at a multiplicity of infection (MOI) of 10, and infected cells were incubated in L-15 medium with 2% FBS at 27 °C for three days until cytopathic effect (CPE) was observed. After CPE was observed, the culture supernatant was collected and centrifuged at 12,000 rpm for 5 min at 4 °C and then kept as the virus stock. To further collect cell lysate, 2 mL of PBS were used to wash cells, and the virus-infected cells were then treated with lysis buffer (150 mM NaCl, 1 mM EDTA, 50 mM Tris-HCl, 0.1% NP-40, and 0.02% protease inhibitor, pH 8). After slow shaking for 30 min at 4 °C, the cell lysate was collected in a 1.5 mL microcentrifuge tube, sonicated for 2 min, and then centrifuged at 12,000 rpm for 10 min at 4 °C. The supernatant was collected in a new 1.5 mL microcentrifuge tube and stored at −20 °C.

### 2.4. Grouper Tissue Lysates Preparation

Uninfected one-year-old orange-spotted groupers were used in the current study; optic nerves were collected and stored in liquid nitrogen. The collected tissue was transferred to a pre-cooled mortar and pestle, and 1 mL of lysis buffer was then added for careful homogenization on ice. The sample was then transferred to a 1.5 mL microcentrifuge tube and stored at −20 °C.

### 2.5. SDS-PAGE

A discontinuous electrophoresis buffer system with a 4% stacking gel and 12% resolving gel was used for protein separation. All samples were boiled for 10 min after the addition of sample loading buffer and subsequently electrophoresed at a voltage of 80 V for the stacking gel and 120 V for the resolving gel until the bromophenol blue reached the bottom of the gels. Protein bands were visualized by staining with Coomassie Brilliant Blue R-250.

### 2.6. Western Blot Analyses

For Western blotting analyses, proteins separated by SDS-PAGE were transferred onto a polyvinylidene difluoride (PVDF) membrane (Merck Millipore) by semi-dry blotting. Membranes were blocked in 5% skim milk (Difco Laboratories, Sparks, MD, USA) in TBS (0.2 M NaCl and 50 mM Tris-HCl, pH 7.4). Immunodetection was performed by incubating the blot in rat anti-NNVCP protein serum diluted 1:10,000 in TBS with 5% skim milk for 1 h at room temperature. Subsequently, goat anti-rat IgG antibody conjugated with horseradish peroxidase (Sigma-Aldrich, St. Louis, MO, USA) was used at a concentration of 1:10,000, and detection was performed with a Western Blot Chemoluminescence Reagent (NEN Life Sciences, Boston, MA, USA). 

### 2.7. Immunoprecipitation (IP) Assay

Native NNVCP was produced by NNV-infected GF-1 cells. The virus infection and cell lysate collection methods were described previously. After the cell lysate was collected, 500 µL of cell lysate and 1 µL of antibody were thoroughly mixed and incubated overnight at 4 °C under gentle agitation or rotation. 30 µL of Protein A agarose beads were added to the mixture and incubated for 4 h at 4°C by gentle mixing on a suitable shaker. After incubation, 300 µL of grouper tissue supernatant were added and incubated overnight at 4 °C on a shaker. 1 mL of lysis buffer was added to the mixture by maintaining gentle agitation and then centrifuged at 6000 rpm for 3 min at 4 °C. Lastly, the supernatant was discarded, and the proteins captured by Protein A beads were preliminarily identified by Western blot. The preliminary identified proteins were further analyzed and annotated by 1DLC, LTQ-Orbitrap MS (BIOTOOLS, New Taipei City, Taiwan). The proteins combined with Protein A beads as a control group.

### 2.8. CK Amino Acid Sequence Analyses and Phylogenetic Construction

The CK complete coding sequence was compared with the GenBank database using the program BlastX. Amino acid sequences of six CK B-type and six M-type isolated from other fish animals were retrieved from GenBank, and were used for sequence alignment and phylogenetic analyses. The complete coding sequences of these CK were subsequently subjected to phylogenetic analyses and were performed with Mega4.0 software using the neighbor-joining algorithm. One thousand bootstrap replicates were generated to test the robustness of the trees. Sequences used in the alignment analyses and phylogenetic tree, followed by their GenBank accession number, were *Poecilia reticulata* (XM_017302257.1), *Xiphophorus maculatus* (XM_023348108.1), *Takifugu rubripes* (XM_003971402), *Stegastes partitus* (XM_008298910.1), *Danio rerio* (XM_005156643.4), and *Oreochromis niloticus* (XM_005453435.4). 

### 2.9. Far-Western Blot Assay

Rabbit anti-Ckba antibody which is specific to CK B-type was bought from a commercial company (GeneTex, Irvine, CA, USA). The target proteins were separated by SDS-PAGE, transferred to a PVDF membrane, and renatured gradually at 4 °C overnight in HEPES buffer (20 mM HEPES, 100 mM NaCl, 1 mM EDTA, 1 mM dithiothreitol, 0.1% Tween 20, 10% glycerol, pH 7.5) containing 5% skim milk. The blot was washed and incubated with 40 μg binding protein (rCKB protein) in 10 ml incubation buffer (20 mM Tris/HCl, 150 mM NaCl, 0.05% Tween 20, 3% skim milk, pH 7.5) for 2 h at room temperature. Then, the blot was incubated in a rabbit anti-Ckba antibody of binding proteins diluted 1:5000 in TBST with 5% skim milk for 1 h at room temperature. Subsequently, goat anti-rabbit IgG antibody conjugated with horseradish peroxidase (Jackson ImmunoResearch, West Grove, PA, USA) was used at a concentration of 1:5000 and detection was performed with a Western Lightning™ Plus-ECL (Bioman, New Taipei City, Taiwan).

### 2.10. Cellular Colocalization of NNVCP and CKB by Indirect Immunofluorescence Assay

GF-1 cells were first transfected with pcDNA3-CKB for two days and then infected with NNV for one day. After transfection and infection, GF-1 cells were rinsed three times with PBS and then were fixed in paraformaldehyde (4% in PBS) for 10 min at 4 °C. After fixation, the solution was replaced with buffer (0.1% Triton X-100, 4% paraformaldyhyde in PBS) for 3 min; then, the cells were washed three times with PBS and incubated with blocking buffer (5% bovine serum albumin and 5% normal goat serum in PBS) for 1 h at room temperature. The GF-1 cells were then treated with polyclonal rat anti-NNVCP antibody (1:200 in blocking buffer) and rabbit anti-Ckba (1∶500 in blocking buffer) for 2 h at room temperature. Next, the cells were washed three times with PBST (0.2% Tween-20 in PBS) and reacted with Cy3-conjugated goat anti-rabbit IgG antibodies (1∶1000 in PBS; Jackson ImmunoResearch) and anti-rat antibody (1∶1000 in PBS; Alexa flour 488) at room temperature. Counterstaining of the nucleus was performed with DAPI. After being washed three times with PBST, the cover glasses were wet mounted and the fluorescent signals were examined with a Leica TCS SP5 Confocal Spectral Microscope Imaging System.

### 2.11. Cell Transfection

To study the function of genes in vitro, cell transfection was conducted using transfection reagent Lipofectamine 2000. Briefly, GF-1 cells were seeded in six-well cell culture plates at 60–70% confluence. The next day, cells were transfected by addition of the mixture of Lipofectamine 2000 and plasmids following the manufacturer’s instructions. Six hours after the transfection, the transfection medium was replaced with the fresh normal medium and cells were cultured at 27 °C for further study. Two days after transfection, the cells were infected for three days and were then collected for further study.

### 2.12. RNA Extraction and qRT-PCR Analysis

Tissues (100 μg) were homogenized in 1 μL of TRIzol reagent (Thermo Fisher Scientific) and then subjected to 2-propanol extraction and ethanol precipitation according to the manufacturer’s recommendations. Total RNA was centrifuged in 75% ethanol at 14,000× *g* for 30 min at room temperature. The pellet was dissolved in DEPC-water and quantified by spectrophotometry. After RNA extraction, 1 μg of total RNA was used for cDNA synthesis using HiScript I Reverse Transcriptase (BIONOVAS, Toronto, ON, Canada). Reverse transcription was conducted according to the manufacturer’s protocol with an Oligo (dT)18 primer. The synthesis condition of cDNA was set at: 65 °C for 5 min, 42 °C for 60 min, and 70 °C for 15 min. Quantitative real-time PCR (qPCR) was performed using the Applied Biosystem^TM^ 7500 Real-Time PCR System (Applied Biosystems, Waltham, MA, USA) on a TOptical Thermocycler ® (Analytik Jena AG, Jena, Germany). The qPCR reaction contains 1 μL of the cDNA template, 10 μL of the 2× qPCRBIO syGreen Master Mix, and 0.8 μL each of the forward and reverse primer (10 pmol/uL) with the following primer set: CKB-qPCR-F/CKB-qPCR-R: (5′-GACACCCAGTGGATTTACTC-3′/5′-GTCCAGCAGCTCTTTGAAGA), NNVRNA2-qPCR-F/NNVRNA2-qPCR-R: (5′-TGTCGCTGGAGTGTTCG-3′/5′-GAAGTCATTTGTGGAAAGGGAATC-3′). The amplification condition was initial denaturation at 95 °C for 5 min, followed by 40 cycles of 95 °C for 5 s, then 65 °C for 30 s. The melting curve and cooling were performed at the last step of qPCR. The primers used in this study were listed in Table 1. The expression levels of the target gene were normalized to beta-actin, a housekeeping gene. Fold change in the relative gene expression with control group was determined by the standard 2^−ΔΔCt^ method. The changes were analyzed by unpaired sample *t*-test. Statistical significance was accepted at *p* < 0.05, and high significance was accepted at *p* < 0.01. All data were expressed as mean ± standard deviation (mean ± SD).

## 3. Results

### 3.1. Identification of Optic Nerve Proteins Interacting with Native NNV Capsid Protein (NNVCP) by Proteomic Analysis of Immunoprecipitation (IP) Assay

**Figure 1:**
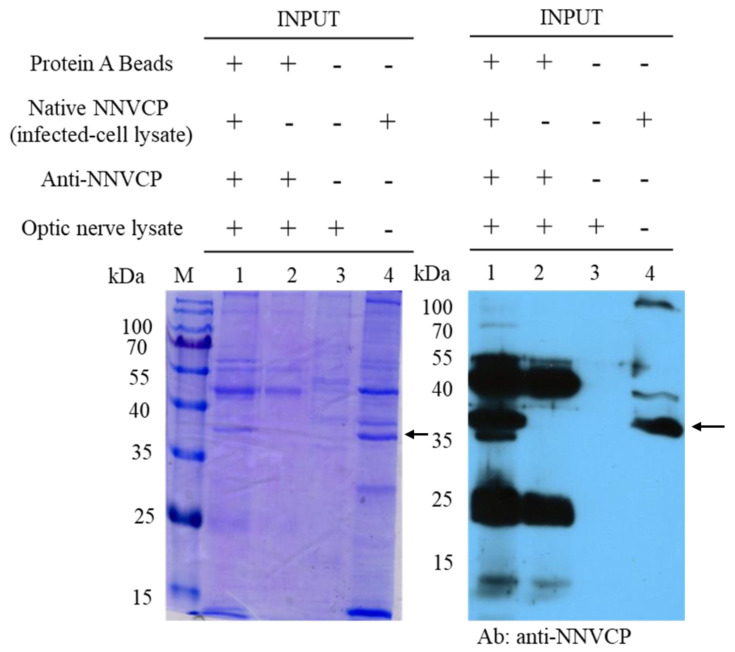
Nervous necrosis virus capsid protein (NNVCP) interactions with grouper optic nerve proteins. SDS-PAGE and Western blot analysis of the grouper proteins that interact with NNVCP by immunoprecipitation (IP) assay. The sample was loaded in every well (listed above the gel) and then detected with the NNVCP antibody. Each well was loaded with 20 µg of protein. Lane 1: lysates from grouper optic nerve and native NNVCP were subjected to immunoprecipitation with Protein A beads. Lane 2: lysates from grouper optic nerve were subjected to immunoprecipitation with Protein A beads (negative control). Lane 3: lysates from non-infect grouper optic nerve. Lane 4: Native NNVCP from infected-cell lysate. The arrow indicates native NNVCP.

To identify host proteins interacting with native NNVCP, the protein–protein interaction assay was performed using an IP assay with natural NNVCP and grouper optic nerve tissue. Protein A beads conjugated with anti-NNVCP specific antibody were first incubated with native NNVCP produced from NNV-infected GF-1 cells and then co-incubated with lysates from grouper optic nerve tissue (Figure 1, lane 1). In the control group, Protein A beads conjugated with anti-NNVCP specific antibodies were incubated directly with the lysate of the optic nerve tissue without adding native NNVCP (Figure 1, lane 2). Three obvious bands were detected in Figure 1: 37 kDa is native NNVCP (Figure 1, lane 1 and lane 4), 25 kDa is the light chain of anti-NNVCP specific antibody, and 50 kDa is the heavy chain of anti-NNVCP specific antibody (Figure 1, lane1 and lane 2). Lane 3 revealed that grouper optic nerve was non-infected NNV. After IP, samples were preliminary evaluated by SDS-PAGE and Western blot. Through mass spectrometry (MS) analysis of optic nerve IP cleavage samples, it was identified that many grouper proteins could interact with NNVCP, and the proteins were analyzed and annotated (Table 1 and Table 2). The score in Table 2 indicates the protein score (MudPI score), which is the sum of the peptide score [26]. MS analysis of the grouper optic nerve protein that can interact with NNVCP found 287 peptides belonging to 49 proteins. Among the proteins involved in multiple functional categories, 12 are ribosomal proteins, 7 are involved in immunity including heat shock protein 60 and heat shock protein 90, 7 are involved in glucose metabolism and ATP generation (such as creatine kinase), 7 are related to the cytoskeleton, 5 are Ca^2+^ binding proteins, 2 are ion regulation proteins, 3 are involved in lipid metabolism, 2 are involved in apoptosis, and 2 are involved in protein hydrolysis. The functions of hemoglobin α chain and Hnrpa01 protein are not very clear (Table 1).

### 3.2. Sequence Analysis of Grouper Creatine Kinase (CK) Protein and Phylogenetic Construction

**Figure 2:**
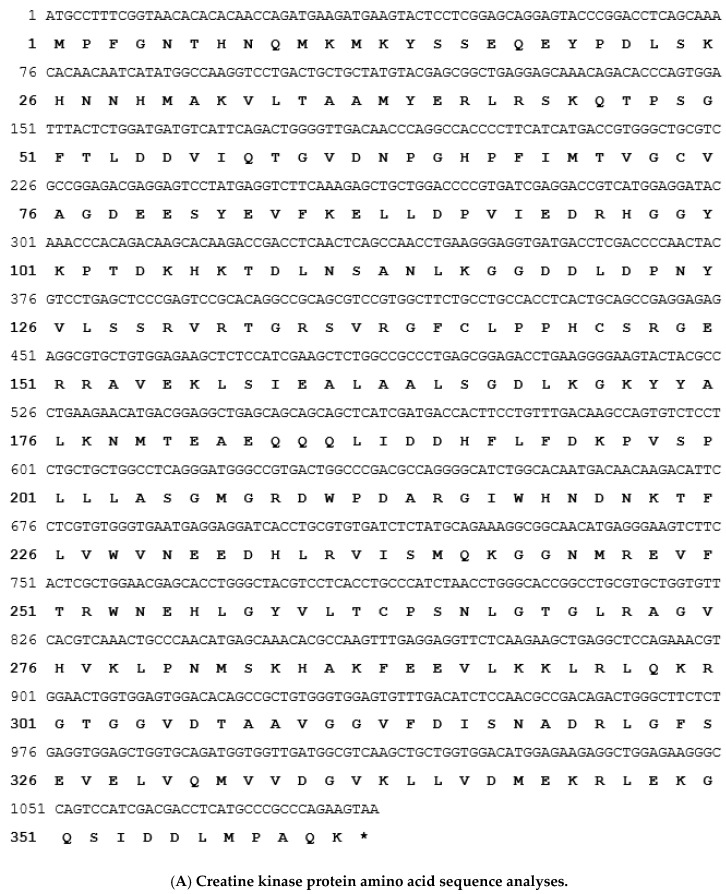

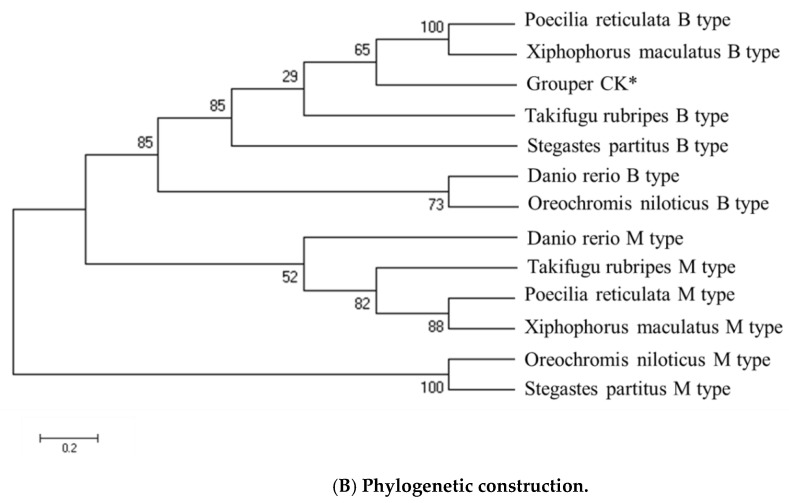
The sequence and phylogenetic analysis of grouper CK. The sequence and phylogenetic analysis of grouper CK. (**A**) the nucleotide sequence and deduced amino acid sequence. (**B**) The phylogenetic tree was constructed by the neighbor-joining method and shows relatedness of 12 CK fish organisms. Robustness was tested by 1000 bootstrap replications and the indicated distance given the value of 0.1 means the 10% differences in amino acid residues between compared sequences. Bootstrap values are given in percentages. * represents the grouper CK sequence used in this study.

According to the EST database, an unpublished sequence with an opening reading frame (ORF) was identified. This ORF encodes a protein of 361 amino acids with a theoretical size of about 42 kDa. The nucleotide sequence surrounding the methionine start codon (GCCATG) of the predicted protein conformed to the Kozak rule of an efficient context for eukaryotic translation initiation. A polyadenylation signal (AAGTAA) was located 1147 bp downstream of the translational stop codon. The deduced amino acid sequence of the ORF was homologous with known grouper CK protein (Figure 2A). A phylogenetic tree was constructed by analyzing the amino acid sequence of grouper CK and the 12 CK protein. The results indicated that grouper CK protein identified to a high degree with those of similar genes in six fish species CK brain type (CKB) compared with CK muscle type (CK M) (Figure 2B).

### 3.3. CKB Interacted with NNVCP In Vitro

**Figure 3:**
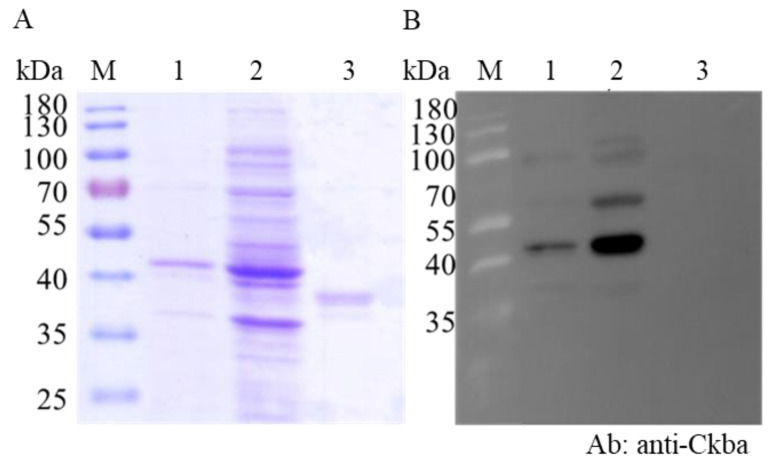

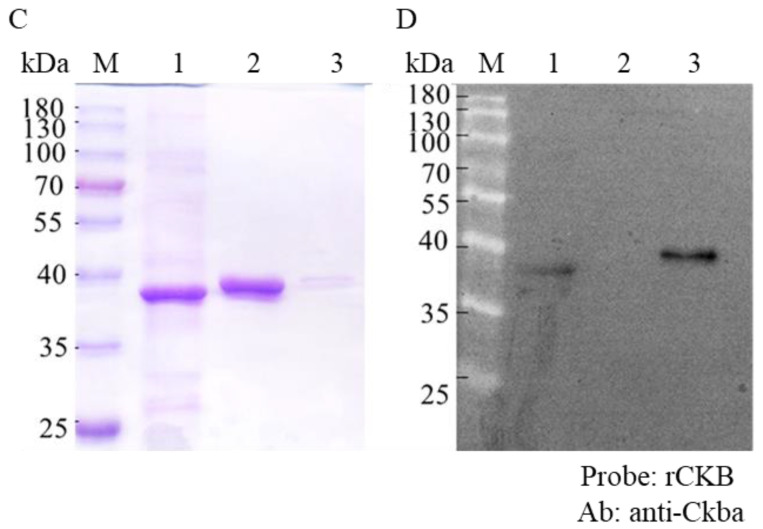
rCKB interacts with rNNVCP in vitro. rCKB interacts with rNNVCP in vitro. (**A**) SDS-PAGE and (**B**) Western blotting were used to determine the recombinant CK B-type (rCKB) generated by *Escherichia coli*. Lane M: pre-stained molecular weight marker. Lane 1: purified rCKB. Lane 2: rCKB in *E. coli* whole lysate. Lane 3: purified rMBP. rCKB protein can be specifically recognized by anti-Ckba antibody. (**C**) SDS-PAGE and (**D**) far-Western blotting were used to determine the interaction between rCKB and rNNVCP. Lane M: marker. Lane 1: rNNVCP. Lane 2: rMBP. Lane 3: rCKB. PVDF membrane was first incubated with rCKB (probe) and then reacted with anti-Ckba antibody. Each well was loaded with 20 µg of protein. rMBP was treated as negative control.

To confirm the interaction of CKB with NNVCP, far-Western blot assay was applied to check the protein–protein interaction in vitro. Recombinant CKB (rCKB) and NNVCP (rNNVCP) were generated by the *Escherichia coli* expression system, and shrimp maltose binding protein (rMBP) was used as a negative control [27]. The Western blot revealed that rCKB protein could be specifically recognized by anti-Ckba antibody (Figure 3B, lane 1 and 2, 42 kDa). However, rMBP could not be recognized by anti-Ckba antibody (Figure 3B, lane 3, 38 kDa) In the far-Western blot analysis, after hybridizing with rCKB and reacting with anti-Ckba antiserum, rCKB showed that it could interact with rNNVCP (Figure 3D, lane 1). As a negative control, rMBP could not interact with rCKB (Figure 3D, lane 2). The results indicated that CKB can interact with NNVCP.

### 3.4. CKB Colocalized with NNVCP in the GF-1 Cell Line

**Figure 4:**
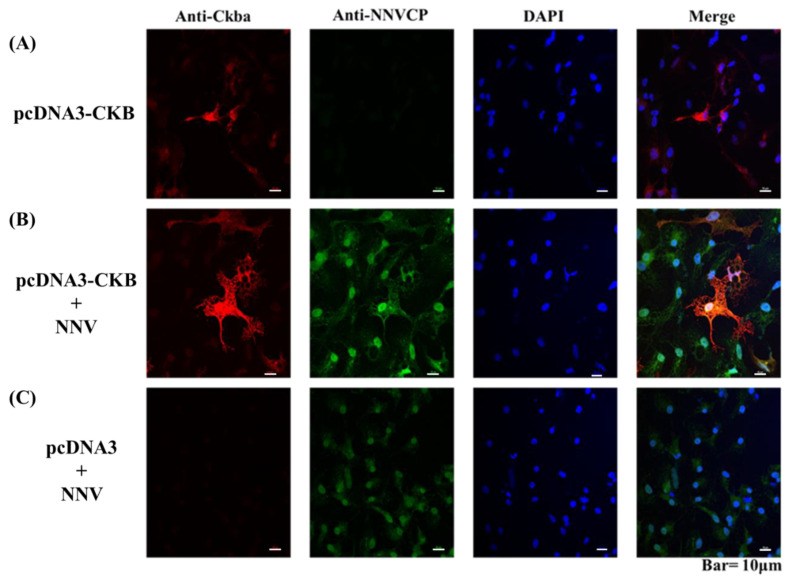
The colocalization between CKB and NNVCP in the GF-1 cell by confocal microscopy. The colocalization between CKB and NNVCP in the GF-1 cell by confocal microscopy. The red fluorescence represents CKB, the green fluorescence represents NNVCP, and the blue fluorescence DAPI represents the nucleus. Colocalization of CKB and NNVCP is indicated in yellow in this merged. (**A**) GF-1 cell transfected with pcDNA3-CKB. (**B**) GF-1 cell transfected with pcDNA3-CKB and infected NNV. (**C**) GF-1 cell transfected with pcDNA3 and infected NNV. Bar = 10 μm.

The interaction of CKB and NNVCP was further validated in vivo by the GF-1 cell line. Since GF-1 cells do not express CKB, this experiment was carried out by transfecting the cells with CKB-pcDNA3 plasmid and then infecting them with NNV. In previous studies, it was found that CKB is distributed throughout the cell, but mainly in the perinuclear area [28]. 48 hours after transfection, the same phenomena were observed in GF-1 cells. CKB proteins were observed in the perinuclear area of GF-1 cells transfected with pcDNA3-CKB (Figure 4A, red fluorescent signals). However, no immunofluorescent signal was detected in GF-1 cells transfected with pcDNA3 only (Figure 4C). Two days after transfection, the cells were infected with NNV. After infection with NNV for 24 h, NNVCP signal was predominantly detected at the nucleus of GF-1 cells (Figure 4B,C, green fluorescent signals). As shown in Figure 4, the immunofluorescent signal of CKB was enhanced after NNV infected the GF-1 cells transfected with pcDNA3-CKB. Furthermore, CKB colocalized with NNVCP in the perinuclear area of NNV-infected GF-1 cells (Figure 4B, Merge).

### 3.5. CKB Participates in NNV Morphogenesis

**Figure 5:**
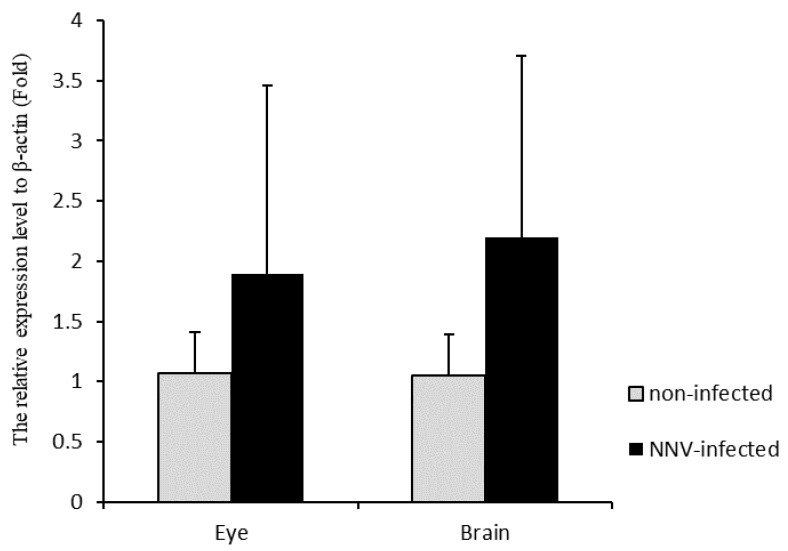
Quantitative real-time PCR analysis of *CKB* gene expression levels between NNV-infected and non-infected tissues. Quantitative real-time PCR analysis of CKB gene expression levels between NNV-infected tissue and non-infected tissue. The relative expression of CKB was normalized to β-actin. Vertical bars represent the mean ± S.D. (*n* = 4).

**Figure 6:**
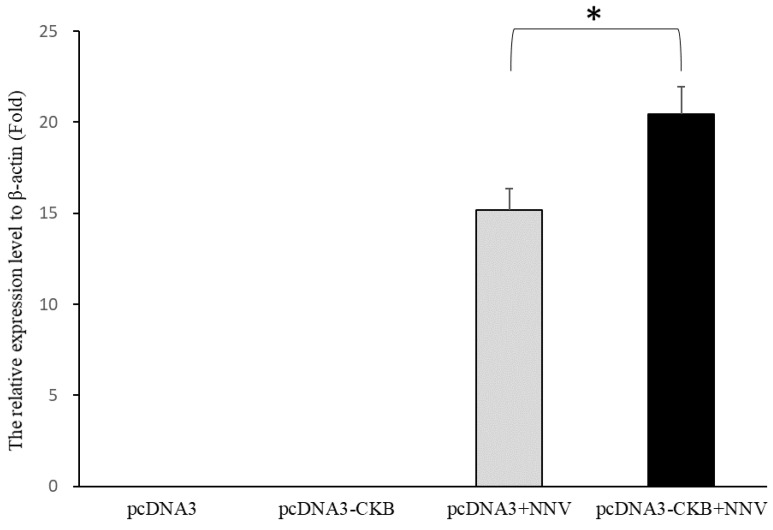
The expression levels of NNVCP gene levels in GF-1 cells after NNV infection. The expression levels of NNVCP gene were measured by quantitative real-time PCR. The relative expression of the NNVCP gene was normalized to β-actin. Significance was measured using the Student’s *t*-test. The data are expressed as the mean ± S.D. (*n* = 4). An asterisk (*) indicates a significant difference between the experimental and control group. * *p* < 0.05.

To verify whether CKB is involved in NNV morphogenesis, we compared eye and brain tissues that were not infected with NNV and infected with NNV. The result showed that CKB expression was significantly upregulated in infected tissues compared to the non-infected tissues. The gene expression of CKB upregulates approximately twofold after NNV infection (Figure 5). To further verify whether CKB does contribute to virus replication, the amounts of NNV virus in GF-1 cells transfected with and without CKB were compared. The results showed that the amount of virus in GF-1 cells transfected with pcDNA3-CKB was significantly higher than that in cells transfected with pcDNA3 only (Figure 6). The mRNA expression level of NNVCP in the pcDNA3-CKB group was 5.29 times higher than that in the control group (pcDNA3 only). Based on the above experiments, the results show that CKB does help NNV morphogenesis.

## 4. Discussion

Nervous necrosis virus (NNV) infection causes high mortality in various economically important fish species worldwide. In recent years, many studies on NNV have been reported, but its infection mechanism remains unclear. In order to accomplish virus morphogenesis, the viral structural protein needs to complete a series of common tasks. Those tasks include recognition and binding to host receptors and co-receptors, entry into the cell by endocytosis or membrane fusion, disassembly of the viral capsid structure, and release of the viral genome. Then, newly viral proteins begin packaging the viral genome and assembling into capsid structures, and finally, mature virions exit the host cell. As the only structural protein, NNV capsid protein (NNVCP) should responsibly serve multiple functions in NNV morphogenesis. In this study, immunoprecipitation (IP) was used to analyze the proteins that bind to NNVCP in optic nerve tissues of grouper (*Epinephelus coioides*) and 49 proteins were identified (Table 1). Several host proteins in our database have been previously confirmed to interact with NNVCP, such as heat shock protein 90 [24] and voltage-dependent anion selective channel protein 2 (VDAC2) that are regarded as required in NNV infection [25]. After comparing the control group, several proteins were excluded from the table, such as histone and HSC70 (Table 2, gray font) [23,29]. Therefore, some proteins that can really bind to NNVCP may be considered as non-specific binding proteins and be eliminated during the screening process. Furthermore, many previous studies found that interferon can inhibit NNV infection [30,31,32,33]. However, no interferon was identified in this study; this may be related to the experimental mechanism in which the grouper itself does not have an immune mechanism that produces interferon. 

The first step for viruses to complete their life cycle is to bind to host cells, which is determined by the structure of the virus itself and the surface composition of host cells. In 49 optic nerve proteins, the possible candidate receptor may be heat shock protein (HSP). HSC70 and HSP90 have been known as a functional part of the NNV receptor complex, and knockdown HSC70 or HSP90 gene expression could reduce the NNV entry [23,24]. Furthermore, many functions of heat shock proteins were described in different kinds of viruses, such as the entry of rotavirus [34], uncoating of adenovirus [35], viral gene transcription of human immunodeficiency virus type 1 (HIV-1) [36], viral genome replication of hepatitis B virus (HBV) [37,38,39], and viral capsid assembly of polyomavirus [40]. Three HSPs (HSP60, HSC70, and HSP90) were screened in this study. Apolipoproteins were also found in our results. Apolipoproteins play an important role in hepatitis C virus (HCV) pathogenesis, including in viral attachment, entry, and assembly [41]. They may not only participate in NNV entry, but also involve other NNV morphogenesis stages. 

As the only structural protein of NNV, NNVCP not only participates in the virus entry stage, but also participates in other stages of morphogenesis. Several proteins that were abnormal in dementia or neurological disorders, such as cytoskeleton and nerve proteins, were found to interact with NNVCP. The eukaryotic cytoskeleton is composed of actin microfilaments, microtubules, and intermediate filaments (IFs), which control cell shape, locomotion, intracellular organization, and transport [42]. Keratins are expressed in most epithelial cells, but keratin 8 (KRT8) and keratin 18 (KRT18) are only expressed in hepatocytes [43]. KRT8 is involved in the replication of HBV DNA and was found to enhance HBV replication [44]. In some neurodegenerative diseases such as Alexander’s disease and Alzheimer’s disease, keratin and desmin aggregates and inclusions can be observed [45,46], and dysregulated keratin expression was observed in Alzheimer’s disease [47]. Alexander’s disease is an early-onset childhood pathology with clinical symptoms including sudden paralysis, convulsions, and stunting that eventually lead to death, with glial fibrillary acidic protein (GFAP), HSP, and αB-crystallin forming aggregates and inclusion bodies in the astrocyte cytoplasm. Ran and Ran-binding proteins are involved in many diseases. Ran-binding protein 2 mutation will cause a familial form of acute necrotizing encephalopathy, a neurological disorder [48], and Ran-binding protein 9 will have large accumulation in the brains of patients with Alzheimer disease; it is thought to be the generation of the toxic neuropeptide amyloid beta and enhance mitochondrial-mediated apoptosis [49,50]. Aldehyde dehydrogenase protein (ALDH) expression level was decreased in the substantia of Parkinson’s disease patients and Alzheimer’s disease [51]. Classical swine fever virus (CSFV) inhibits INF expression by binding to hemoglobin and also inhibits INF inhibition when hemoglobin is overexpressed [52]. In HIV patients, low hemoglobin levels were associated with higher interferon and lower transferrin, which may cause anemia in these patients [53]. NNVCP binding to hemoglobin and transferrin may cause anemia upon grouper infection with NNV. Collectively, those related proteins were also confirmed to bind to NNVCP in IP database. This result may explain the abnormal behavior, vacuolation, and neuronal degeneration of the brain and retina and anemia in groupers infected with NNV [54], and may be related to necrosis of the central nervous system.

Some calcium-binding proteins were found to interact with NNVCP in the optic nerve tissues, such as calmodulin, ictacalcin, calreticulin, galactoside binding lectin, and S100-A11 calcium-binding protein. Calcium ions (Ca^2+^) play important roles in cell signaling, especially in the endoplasmic reticulum. In Dengue virus-infected cells, the unfolded protein response was associated with Ca^2+^ signaling [55]. The NNV motif _130_DxxDxD_135_ is associated with calcium ions and was found to be crucial for the assembly and stability of NNV particles [56]. Calmodulin (CaM) is a universal intracellular Ca^2+^ receptor that binds to numerous target proteins and modulates their activities in response to Ca^2+^ signals. CaM plays a critical regulatory role in essential biological functions such as metabolism, motility, memory, immune responses, and apoptosis [57,58]. Autophagy is important for the replication of numerous DNA and RNA viruses [59]. Autophagy can be initiated by virus-encoded proteins that release ER calcium into the cytoplasm and activate a CaM signaling pathway, after which the virus uses the autophagy membrane trafficking pathway to transport viral proteins for virus replication; for example, CaM interacts with viral proteins involved in HIV-1 replication [60]. The Enterovirus 71 (EV71) VP1 protein can activate CaMKII and has a structural role in viral replication [61]. The IP results in the present study may indicate that calcium-binding related proteins are involved in NNV replication.

Tropomyosin, myosin, and actin were all identified in this study. Several viruses including murine leukemia virus (MLV) and avian leukosis virus (ALV) have been demonstrated to interact with actin and myosin at the cell entry site, which helps these viruses more efficiently infect the host cell [62]. Blocking the myosin–actin interaction could inhibit HIV virus budding [63]. In addition, the actin–myosin network is important and necessary for influenza virus production. In shrimp virus research, the interaction of tropomyosin and white spot syndrome virus VP466 protein (WSSV-VP466) was found to enhance WSSV infection [64]. Tropomyosin, myosin, and actin were all identified in this study, suggesting that NNV may also use these proteins to help virus invasion of host cells, transport within the cells, or virus budding. Additional experiments are needed to investigate this hypothesis.

Virus replication requires energy and macromolecule synthesis, and host cells provide the viruses with metabolic resources necessary for their efficient replication. Thus, it is highly likely that interaction of viruses with key molecules in host cell metabolic pathways, including energy-generating systems, contributes to the virus replication. In the previous study, the ATP level in NNV-infected cells was lower than that in non-infected cells; that means that NNV replication depleted host ATP [25]. Mitochondrial creatine kinase (MtCK), adenine nucleotide translocase (ANT), and VDAC2 formed a complex in humans [65]. In the IP interaction database, the proteins that participated in ATP generation, such as ADP/ATP translocase, CK, VDAC1, and VDAC2, were verified. In order to verify that the interaction of NNV with cellular molecule of ATP generation pathway contributes to virus amplification, and also to confirm that the reliability of our IP results is very high, we chose CK for subsequent analysis. CK catalyzes the reversible transfer of the phosphate group of phosphocreatine (pCr) to ADP to yield ATP and creatine and is known to play important roles in local delivery and cellular compartmentalization of ATP [66]. CK also plays an important role in tissues that require a lot of energy, such as muscle, optic nerve, and brain, in which the brain and optic nerve are the major infectious tissue of NNV [67]. Furthermore, VDAC2 is required for NNV infection for maintaining the cellular ATP level and has a positive regulate on virus-induced apoptosis [25]. Since VDAC2 has been shown required when NNV infection, and based on the indirect relationship between VDAC2/CK/NNVCP, we further validate the interaction relationship between CK and NNVCP. The CKB-NNVCP complex was clearly detected in vitro using anti-Ckba antibody in far-Western blot assay (Figure 3D), and the in vivo immunostaining assay revealed that CK was colocalized with NNVCP (Figure 4B). In addition, we demonstrated that the presence of CKB could enhance NNV replication (Figure 6). Therefore, we concluded that NNVCP, through its interaction with CKB, controls the generation of ATP in the host cell to enhance virus replication.

In summary, in this research, according to IP experiment data, we first identified 49 grouper optic nerve proteins involved in NNV morphogenesis by interacting with NNVCP. In NNV-infected tissue, CKB gene expression was upregulated; meanwhile, NNV replicate will enhance by interacting with CKB. Here, CKB was identified as a novel enhancer for NNV through interact with NNVCP. Our findings would be beneficial to extend to an understanding of the host cell response and morphogenesis of NNV. This may lead to the development of a new type of antiviral agent.

## Figures and Tables

**Table 1:** Mass spectrometry (MS) analysis of groupers optic nerve proteins that can interact with NNVCP by immunoprecipitation (IP).

Protein Functional Classification and Description	
**Ribosomal protein**	
ribosomal protein S7	60S ribosomal protein L30
ribosomal protein LP0	ribosomal protein S13
ribosomal protein L7	60S ribosomal protein L27
ribosomal protein L23	40S ribosomal protein S2
60S ribosomal protein LP1	60S ribosomal protein L13
60S ribosomal protein L31	40S ribosomal protein S18
**Immunity**	
heat shock protein 60	immunoglobulin mu heavy chain
immunoglobulin light chain	natural killer cell enhancement factor
heat shock protein 90 immunoglobulin light chain	immunoglobulin heavy chain variable region
**Glucose metabolisms and ATP generation**	
ADP-ATP translocase	creatine kinase
transferrin	tryptase-2 precursor
nucleoside-diphosphate kinase	triosephosphate isomerase B
fructose-bisphosphate aldolase A	
**Cytoskeleton proteins**	
keratin type II E3	tropomyosin α-4 chain
elongation factor 1-α	myosin light chain 3
eukaryotic translation elongation factor 2	tropomyosin
smooth muscle cell-specific protein SM22 α	
**Ca^2+^ binding protein**	
calmodulin	S100-like calcium binding protein
ictacalcin	calreticulin
galactoside binding lectin	
**Ion regulation protein**	
voltage-dependent anion selective channel protein 2	voltage dependent anion channel protein 1
**Lipid metabolism proteins**	
apolipoprotein AI	brain-type fatty acid binding protein
14 kDa apolipoprotein	
**Apoptosis protein**	
glyceraldehyde-3-phosphate dehydrogenase	ran protein
**Protein hydrolysis**	
aldehyde dehydrogenase family 9	lactate dehydrogenase-A
**Other**	
hemoglobin α chain	Hnrpa01 protein

**Table 2:** The original protein assay data in the grouper optic nerve with native NNVCP by immunoprecipitation. The gray font indicates the proteins that were also detected in the control group.

GenBank Accession No.	Description	Score *
AAR97600.2	beta actin ( *Epinephelus coioides* )	2492.00
3JBM	C Chain C, Virus-like Particle of Orange-spotted Grouper Nervous Necrosis Virus	1706.91
AER42656.1	keratin 8, partial (*Epinephelus coioides*)	1457.49
AAX78203.1	immunoglobulin mu heavy chain (*Epinephelus coioides*)	1263.15
AAX78206.1	immunoglobulin mu heavy chain (*Epinephelus coioides*)	999.30
AGG55392.1	voltage-dependent anion selective channel protein 2 (*Epinephelus coioides*)	944.17
AAX78208.1	immunoglobulin mu heavy chain (*Epinephelus coioides*)	914.30
AEG78351.1	keratin type II E3, partial (*Epinephelus coioides*)	790.40
ACM48181.1	apolipoprotein AI, partial (*Epinephelus coioides*)	745.80
AGG55391.1	heat shock cognate protein 70 (*Epinephelus coioides*)	745.79
ACH73075.1	keratin 8, partial (*Epinephelus coioides*)	740.04
AIS72878.1	heat shock protein 60 (*Epinephelus coioides*)	679.74
AEW43726.1	transferrin (*Epinephelus coioides*)	645.54
AER42652.1	hemoglobin beta chain (*Epinephelus coioides*)	623.08
ADG29138.1	tropomyosin (*Epinephelus coioides*)	539.59
ABW74631.1	immunoglobulin light chain (*Epinephelus coioides*)	504.86
AHB51756.1	calmodulin (*Epinephelus coioides*)	491.05
ABW04131.1	glyceraldehyde-3-phosphate dehydrogenase, partial (*Epinephelus coioides*)	484.23
AAS55942.1	immunoglobulin light chain variable region (*Epinephelus coioides*)	476.38
ABW04145.1	smooth muscle cell-specific protein SM22 alpha (*Epinephelus coioides*)	430.16
AEO89322.1	voltage dependent anion channel protein 1 (*Epinephelus coioides*)	421.60
AER42688.1	tropomyosin alpha-4 chain, partial (*Epinephelus coioides*)	418.42
ABW04135.1	natural killer cell enhancement factor (*Epinephelus coioides*)	361.05
AEG78406.1	hemoglobin alpha chain, partial (*Epinephelus coioides*)	347.41
ADG29126.1	fructose-bisphosphate aldolase A (*Epinephelus coioides*)	340.73
AEG78409.1	60S ribosomal protein L30 (*Epinephelus coioides*)	325.27
ADG29180.1	triosephosphate isomerase B (*Epinephelus coioides*)	310.40
ACL98136.1	type I keratin, partial (*Epinephelus coioides*)	305.82
ABW04143.1	S100-like calcium binding protein (*Epinephelus coioides*)	298.82
ABW04124.1	ADP-ATP translocase (*Epinephelus coioides*)	288.91
ACH73065.1	ribosomal protein S7 (*Epinephelus coioides*)	283.43
ADZ76534.1	myosin light chain 3 (*Epinephelus coioides*)	279.24
ABW04132.1	Hnrpa01 protein, partial (*Epinephelus coioides*)	269.70
ACV04938.1	heat shock protein 90 (*Epinephelus coioides*)	269.27
ADG29156.1	histone H2B (*Epinephelus coioides*)	265.26
AOW69105.1	elongation factor 1-alpha (*Epinephelus coioides*)	264.68
ADG29136.1	beta-enolase (*Epinephelus coioides*)	247.11
AEG78428.1	60S ribosomal protein L10a (*Epinephelus coioides*)	246.47
AAW29021.1	lactate dehydrogenase-A, partial (*Epinephelus coioides*)	212.87
ABW04139.1	ribosomal protein LP0 (*Epinephelus coioides*)	212.25
ABW04136.1	nucleoside-diphosphate kinase (*Epinephelus coioides*)	199.15
ADG29150.1	60S ribosomal protein L19, partial (*Epinephelus coioides*)	186.15
ADG29176.1	muscle phosphoglycerate mutase 2 (*Epinephelus coioides*)	184.01
ACH73061.1	ribosomal protein L7, partial (*Epinephelus coioides*)	157.36
ABW04127.1	brain-type fatty acid binding protein (*Epinephelus coioides*)	156.32
ABW04123.1	60S ribosomal protein L27 (*Epinephelus coioides*)	152.44
ADZ99127.1	immunoglobulin heavy chain variable region, partial (*Epinephelus coioides*)	146.61
AEG78395.1	aldehyde dehydrogenase family 9 member A1-A, partial (*Epinephelus coioides*)	136.08
ADG29178.1	tryptase-2 precursor, partial (*Epinephelus coioides*)	135.49
ACL98142.1	desmin, partial (*Epinephelus coioides*)	120.60
ABW04138.1	ribosomal protein L23, partial (*Epinephelus coioides*)	110.93
ACH73060.1	ictacalcin (*Epinephelus coioides*)	104.23
ABW04130.1	galactoside binding lectin (*Epinephelus coioides*)	100.05
AER42692.1	60S ribosomal protein LP1 (*Epinephelus coioides*)	99.41
AEG78402.1	40S ribosomal protein S18 (*Epinephelus coioides*)	87.32
ADG29169.1	60S ribosomal protein L13, partial (*Epinephelus coioides*)	86.49
ADG29143.1	60S ribosomal protein L31 (*Epinephelus coioides*)	67.76
ACM41841.1	creatine kinase, partial (*Epinephelus coioides*)	58.09
AEG78365.1	40S ribosomal protein S2 (*Epinephelus coioides*)	57.41
AEG78426.1	eukaryotic translation elongation factor 2, partial (*Epinephelus coioides*)	55.83
ABW74647.1	immunoglobulin light chain (*Epinephelus coioides*)	51.54
ACM41842.1	14 kDa apolipoprotein, partial (*Epinephelus coioides*)	50.87
ACL98134.1	ran protein, partial (*Epinephelus coioides*)	48.98
AHA43788.1	calreticulin (*Epinephelus coioides*)	38.35
ABW04141.1	ribosomal protein S13, partial (*Epinephelus coioides*)	32.62

*: MudPI score.
